# Recent developments in animal sciences 

**DOI:** 10.17179/excli2015-325

**Published:** 2015-05-11

**Authors:** Seddik Hammad, Isabelle Sobor, Mohammed F. Abdallah, Ahmed A.A. Abdel-Wareth, Mohammad S. Al-Aboody, Mosaab A. Omar

**Affiliations:** 1 Department of Forensic Medicine and Veterinary Toxicology, Faculty of Veterinary Medicine, South Valley University, Qena-Egypt; 2 Department of Environmental Toxicology, Faculty of Biology, University Essen-Duisburg, Essen-Germany; 3 Department of Pharmaceutical Toxicology, Faculty of Pharmacy, Hacettepe University, Sihyyie/Ankara-Türkiye; 4 Animal Nutrition Group, Institute of Animal Science, University of Bonn, Germany; 5 Department of Medical Laboratories, College of Science, Majmaah University, AlZulfi-KSA

## Dear Editor,

Recently, a new journal, JEAAS (Journal of Experimental and Applied Animal Sciences) has been launched. The goal of this new interdisciplinary journal is to advance the field of animal sciences. A particular focus is the establishment and application of animal models for basic and clinical research (Intzoglou et al., 2014[13]; Graham et al., 2014[7]; Musk et al., 2014[18]; Rezaei et al., 2014[19]; Bénardeau et al., 2013[1]; Deluzurieux et al., 2013[3]). Particularly, the advent of transgenic and knockout technologies together with the generation of humanized animals (Hammad et al., 2013[10]) opens exciting possibilities. A second field of interest is traditional and applied animal sciences including farm animals, animal nutrition and related fields of veterinary medicine (Mallaiah et al., 2014[16]; Soriano-Úbeda et al., 2013[21]; Mishra et al., 2013[17]; Zarrindast et al., 2013[22]; Ikewuchi et al., 2013[12]).

A currently particularly active research field is the establishment of *in vitro* systems for applications in pharmacology and toxicology (Kim et al., 2015[14]; Godoy et al., 2013[6]; Zellmer et al., 2010[23]; Drasdo et al., 2014[4]; Grinberg et al., 2014[8]; Schug et al., 2013[20]; Krug et al., 2013[15]). A limitation of *in vitro* data is that comprehensive studies on the *in vivo *relevance of responses observed *in vitro* are rare (Bolt et al., 2014[2]; Ghallab and Bolt, 2014[5]; Hammad et al., 2014[11]; Hammad, 2013[9]). Therefore, JEAAS particularly welcomes studies that compare mechanisms observed in *in vitro* systems to the corresponding processes in organs or organisms. We hope that JEAAS will play a constructive role in this field of research.
